# Corps étranger post traumatique de la paroi thoracique de diagnostic tardif: à propos d’un cas

**DOI:** 10.11604/pamj.2018.31.242.17622

**Published:** 2018-12-20

**Authors:** Abdourahmane Ndong, Papa Ousmane Ba

**Affiliations:** 1 Service de Chirurgie Générale, Hôpital Régional de Saint Louis, Sénégal; 2 Service de Chirurgie Thoracique et Cardio-Vasculaire de Fann, Dakar, Sénégal

**Keywords:** Corps étranger, traumatisme, métal, Foreign body, trauma, metal

## Abstract

We here report the case of a 63-year old patient with a 6-year history of sutured traumatic sore in the right external chest wall. Examination showed a swelling next to the scar which was soft and painless. Ultrasound showed hyperechogenic structure surrounded by a nonvascularized tissue mass. Resection specimen analysis showed metallic foreign body surrounded by a reactive tissue. Foreign bodies located in the soft parts are mostly post traumatic. Their diagnosis is easily made by clinic examination and imaging tests, especially when trauma is localized. In patients with multiple traumas they are sometimes ignored resulting in delayed diagnosis. Imaging plays a key role in diagnosis.

## Image en médecine

Il s'agit d'un patient de 63 ans, aux antécédents de plaie traumatique de la face externe droite du thorax il y a 6 ans qui a été suturée. L'examen montrait une tuméfaction en regard de la cicatrice qui était molle et indolore. L'échographie réalisée était en faveur d'une structure hyperéchogène entourée d'une masse tissulaire sans caractère vasculaire. L'exérèse réalisée a permis de visualiser à l'ouverture de la pièce un corps étranger métallique entouré d'un tissu réactionnel. Les corps étrangers localisés dans les parties molles sont pour la plupart post traumatiques. Leur diagnostic est aisé avec la clinique et l'imagerie surtout si le traumatisme est localisé. Dans le cadre d'un polytraumatisme, ils peuvent être méconnus et leur diagnostic fait tardivement. L'imagerie garde une place de choix dans le diagnostic.

**Figure 1:**
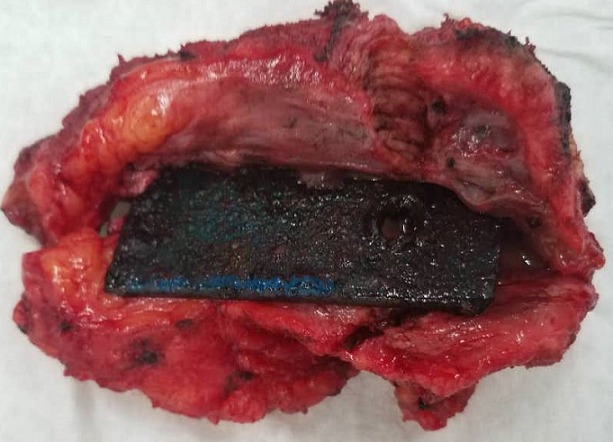
Corps étranger métallique entouré d’un tissu inflammatoire réactionnel

